# Leflunomide Induced Atypical DRESS: A Case Report and Literature Review

**DOI:** 10.31138/mjr.34.1.91

**Published:** 2023-03-31

**Authors:** Maryam Fatima, Salwa Sahar Azimi, Soumya Ashwini, Madhuri H Radhakrishna

**Affiliations:** 1 Clinical Pharmacist-AIG Hospitals, India,; 2 Clinical Pharmacist intern, Department of Pharmacy Practice, G. Pulla Reddy College of Pharmacy, India,; 3 Consultant Rheumatologist-AIG Hospitals, India

**Keywords:** DRESS, life-threatening, drug-induced, herpes viruses, Spanish guidelines, national guidelines, Leflunomide, antirheumatic drug

## Abstract

Drug rash with eosinophilia and systemic symptoms syndrome (DRESS syndrome) is a potentially life-threatening, drug-induced, multi-organ system reaction, the most frequently involved organ is liver, followed by the kidneys and lungs.^1^ Early detection and diagnosis followed by withdrawal of the offending agent is vital to minimise the associated morbidity and mortality. A detailed drug history is vital to identify the causative drugs. Although Spanish guidelines were developed by a panel of allergy specialists from the Drug Allergy Committee of the Spanish Society of Allergy and Clinical Immunology (SEAIC) and are available in literature from 2020, many clinicians are still unaware about the management of this syndrome. Framing national guidelines for the early diagnosis and Pharmaco-therapeutic management of DRESS will help the healthcare professionals to save the patients from unintended vulnerability. Leflunomide, a drug widely used in rheumatology and orthopaedics must be used with caution since it has the potential to cause DRESS syndrome. We report a case of a lady aged 32 years, presented to our hospital with a history of leflunomide intake and symptoms of DRESS.

## INTRODUCTION

DRESS syndrome is a severe, drug-induced, idiosyncratic multisystem reaction to a drug, characterised by fever, skin rash, lymphadenopathy, haematological abnormalities, and internal organ involvement.^2^ The European Registry of Severe Cutaneous Adverse Reactions (RegiSCAR), introduced a diagnostic scoring system for DRESS in 2007.^3^ Leflunomide, a disease-modifying and antirheumatic drug (DMARD) has been very rarely reported as a cause of DRESS syndrome. We report a rare case of a lady aged 32 years, presented to our hospital with a history of leflunomide intake and symptoms of DRESS. This case study highlights the potential risk of leflunomide in causing DRESS syndrome and the poor prognosis associated with the syndrome.

## CASE REPORT:

A 32-year-old lady presented with complaints of a rash with itching for 20 days, fever for 17 days, vomiting and loose stools for 1 day. The rash had started from the legs and spread to the entire body. It was a maculopapular rash with mucosal involvement at the angles of the mouth. On examination, she was lethargic, febrile, and icterus was present. Systemic examination was otherwise normal.

On admission, the laboratory investigation revealed Eosinophilia, atypical Leucocytosis, deranged LFT. CBP results showed predominant leucocytosis with eosinophilia. Peripheral smear and Bone marrow aspiration showed features suggestive of reactive cellular marrow with mild eosinophilia and mild megakaryocytic hyperplasia. CRP was positive with high range. PT with INR was mild high, peripheral smear showed predominantly normocytic hypochromic picture along with few microcytes, Procalcitonin-serum showed severe systemic inflammation (5.53ng/ml), Ultrasound of abdomen on day 1 showed mild hepatomegaly, gallbladder wall oedema, minimal ascites. The detailed laboratory investigations are given in **Table 1**.

**Table 1.** Common drugs causing DRESS.^3^

**Class**	**Drugs**
Antiepileptics	Carbamazepine, lamotrigine, phenytoin
Anti-gout medicines	Allopurinol, febuxostat*
DMARDs/immunomodulatory drugs	Sulfasalazine, dapsone, hydroxychloroquine, leflunomide, azathioprine,^[15]^ daclizumab, solcitinib
Antibiotics	Amoxicillin, ampicillin, trimethoprim-sulfamethoxazole, azithromycin, levofloxacin, minocycline, piperacillin/tazobactam, vancomycin, cephalosporin
NSAIDs	Aceclofenac, celecoxib, ibuprofen, aspirin
Antituberculous medications	Ethambutol, isoniazid, pyrazinamide, rifampin, streptomycin
Others	Atorvastatin, amitriptyline, omeprazole

* Seen in patients with CKD; DMARDs: Disease-modifying antirheumatic drug, NSAIDs: Nonsteroidal anti-inflammatory drugs, CKD: Chronic kidney disease

## DIFFERENTIAL DIAGNOSIS

Upon taking past medication history by the clinical pharmacist, the patient revealed that she had taken Ayurvedic medicines for jaundice, but it did not subside. She consulted a local hospital where she was treated with Doxycycline, Ceftriaxone, Ursodeoxycholic acid, and Ademethionine. She had joint pains for which Aceclofenac, Paracetamol, Rabeprazole, Domperidone, Deflazacort, and Leflunomide was advised for 1 month in suspicion of arthritis, after few weeks of medication intake she developed rashes, recurrent fever spikes with itching, hence she was shifted to our hospital for further management. On literature review, it was found that Doxycycline, certain NSAIDS and Leflunomide had documented DRESS reactions. Based on detailed literature review and results of laboratory investigations, Leflunomide-induced DRESS was considered as a final diagnosis. Our patient had a RegiSCAR score of 7 as detailed in **Table 3**, which makes her a case of definite DRESS Syndrome.

**Figure 1.**
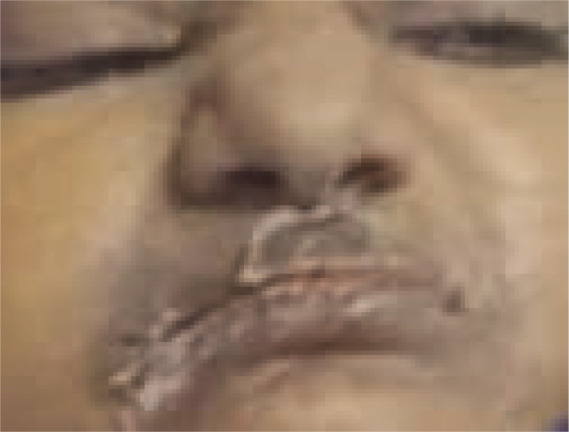
Mucosal involvement of the lesions at the angles of the mouth.

**Figure 2.**
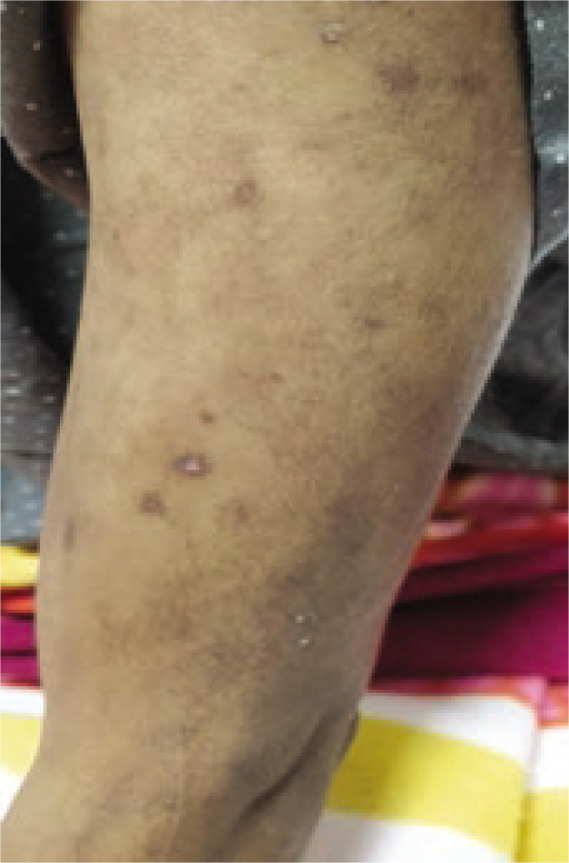
Development of rashes on the arm due to leflunomide.

**Figure 3.**
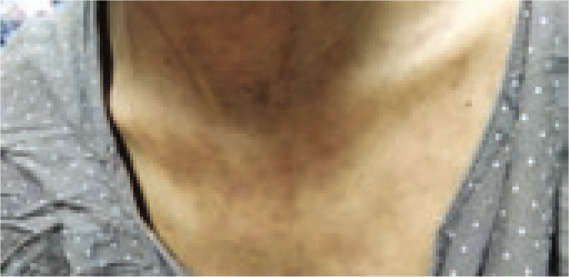
Development of rashes on the neck during the hospital stay.

**Table 2.** Laboratory investigations of the patient during the hospital stay.

**LABORATORY INVESTIGATIONS**	**DAY 1**	**DAY 2**	**DAY 3**	**STEROID THERAPY STARTED**	**DAY 5**	**DAY 8**	**DAY 13**	**DAY 16**	**NORMAL RANGE**
**Complete blood count**									
Haemoglobin	**11.0**	**9.4**	**8.9**		**8.1**	**6.0**	**7.2**	**8.3**	12.0–15.0gm/dl
RBC	**3.7**	**3.3**	**3.1**		**2.8**	**2.2**	**2.7**	**3.1**	4.5–5.5 million/mm^3^
PCV	**30**	**27**	**26**		**23**	**18**	**22**	**25**	40–50%
RDW	13	13	14		14	**15**	**15**	**15**	11.6–14%
Neutrophils (%)	64	61	60		40	50	69	76	40–80%
Lymphocytes (%)	**11**	**18**	31		**45**	25	28	20	20–40%
Eosinophils (%)	**21**	**18**	02		05	**15**	01	02	1–6%
Monocytes (%)	4	**0.3**	7		10	10	02	02	2–10%
Basophils (%)	00	00	00		00	00	00	00	0–2%
Absolute neutrophils count	**7732**	6527	5520		28880	2900	4692	7296	2000–7000 cells/mm^3^
Absolute lymphocyte count	1243	1926	2852		**3240**	1450	1904	1920	1000–3000 cells/mm^3^
Absolute eosinophil count	**2373**	**1926**	184		360	**870**	68	192	20–500 cells/mm^3^
Absolute monocyte count	452	321	644		720	580	136	0	200–1000 cells/mm^3^
Platelet count (x10^3^)	232	219	159		1500	150	254	300	150000–410000/mm^3^
Total WBC	**11300**	**10700**	9200		7200	5800	6800	9600	4000–10000clls/mm^3^
LFT									
Total bilirubin	**8.9**	**9.8**	**10.2**		**10.2**		**12.7**	**9.1**	0.3–1.2 mg/dL
Direct Bilirubin/Indirect Bilirubin	**5.4/**3.5	**6.7/3.7**	**6.3/3.9**		**6.3/3.9**		**7.5/**5.2	**5.1/4.0**	0–0.2mg/dl 0.2–0.8 mg/dL
SGPT (ALT)/SGOT (AST)	**386/583**	**553/993**	**450/650**		**307/281**		**295/294**	**212/162**	Upto 40 U/L/0–35 U/L
ALP	**176**	**152**	**155**		**200**		**394**	**397**	30–120U/L
Total proteins	**5.5**	**4.8**	**5.0**		**5.4**		**5.3**	**5.5**	6.6–8.3g/dl
Albumin	**2.9**	**2.3**	**2.4**		**2.5**		**2.8**	**3.0**	3.5–5.1g/dl
C-reactive protein	**76.1**	**188**				**29**			<10mg/dl
Ferritin		**22550**				**2070**			13–150ng/ml

For the management, we started intravenous steroid therapy (Inj. Hydrocort 50 mg IV Q12H for 5 days followed by Tab. Wysolone 20 mg p/o Q12H) and stopped all previous medications including antibiotics and supportive care was given for liver injury. Cholestyramine was advised for the washout of Leflunomide.

The patient’s LFT and ferritin were improved, other symptoms also subsided including fever, the maculopapular lesions, and itching. Biopsy was advised on follow-up if any of the symptoms reappears. She was discharged on oral steroids and cholestyramine. She did not take cholestyramine and came for follow-up with worsening skin lesions and did not get readmitted, lost to follow-up. On a follow up call, the patient’s attendant informed us about death at home.

## DISCUSSION

DRESS is initiated by pyrexia with body temperature >38°C, early in the course of the disease followed by development of rashes usually maculopapular morbilliform exanthem (usually starting on the face and then generalised), with multiple follicular papules over the body mimicking pityriasis rubra pilaris.^4^ Presence of facial oedema is a characteristic manifestation of DRESS, which mostly occurs with a serious reaction.^5^

The clinical symptoms of DRESS usually occur 2–6 weeks after starting offending drug, which often causes the potential diagnostic delay^4^ or can lead to misdiagnosis by the physician. Early withdrawal of offending drugs is needed once DRESS is identified. The recovery from this condition has been reported to be slow.^5^ Upon rechallenge with the offending drug, symptoms may reappear within 1 day.^5^

Reactivation of HHV-6 is found exclusively in DRESS (role of HHV-6 is unique in DRESS).^6^ However, it was not the case in our patient.

**Figure 4.**
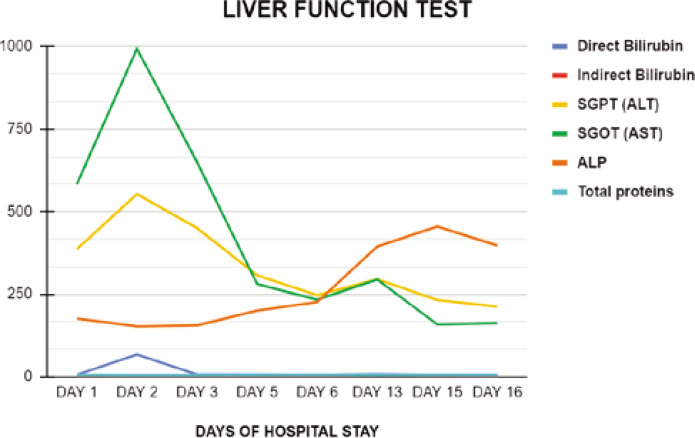
Line chart representing LFT at different days of hospital stay.

The EUROPEAN RegiSCAR decided on a scoring system to help clinicians confirm or exclude the diagnosis of DRESS syndrome.^7^ We evaluated this case using the RegiSCAR criteria that is frequently used for the diagnosis of DRESS.

RegiSCAR Criteriato evaluate DRESS is given in **Table 3**. Our patient had a RegiSCAR score of 7, which makes her a case of definite DRESS Syndrome. Genetic factors are also important in DRESS. Gene polymorphism for drug metabolism enzymes including CYP450 enzymes and N-Acetyltransferase is one risk factor. HLA gene polymorphisms explain the genetic disposition of patients with DRESS.^6^

**Table 3.** RegiSCAR scoring for DRESS Syndrome.^7^

**Criteria**	**No**	**Yes**	**Unknown**	**Case 1**
**Fever >38°C**	−1	0	−1	**0**
**Enlarged Lymph nodes (>2 sites, >1 cms)**	0	1	0	**0**
**Atypical Lymphocytes**	0	1	0	**1**
**Eosinophilia**700–1499 or 10–19.9%	0		0	
		1		
>1500 or >20%		2		**2**
**Skin Rash**	0		0	
Extent >50%	0	1	0	**1**
At least two: Oedema, infiltration, purpura scaling	−1	1	0	**1**
Biopsy suggesting DRESS	−1	0	0	**0**
**Internal Organ involved**	0		0	
One		1		**1**
Two or more		2		
**Resolution >15 days**	−1	0	−1	**0**
**At least three biological investigations done and negative to exclude alternative diagnoses**	0	1	0	**1**
**TOTAL SCORE**				7

DRESS is usually accompanied with internal organ impairment. Haematological changes, eosinophilia is a characteristic feature of the disease. Haematological changes involve eosinophilia and mononucleosis-like atypical lymphocytosis in DRESS with liver as the most common internal organ involved. Renal, cardiac, and lung involvement is common although neurological involvement is rare.^6,9,10^

The management involves early detection, diagnosis followed by prompt withdrawal of the offending agent is vital to minimise the associated morbidity and mortality. Supportive care is recommended including local systemic treatment, systemic steroids to relieve symptoms. Systemic corticosteroids can reduce symptoms of delayed hypersensitivity reactions.^11^ They have also been shown to block the effect of IL-5 on in vivo eosinophil accumulation.^12^ Relapses have also been identified in many case reports, after tapering or withdrawal of systemic steroids^13^, which further emphasizes their role in patients with DRESS syndrome. The mainstay of treatment is the use of topical and systemic corticosteroids, but other options such as intravenous immunoglobulin, cyclosporine, mycophenolate mofetil, rituximab, and cyclophosphamide have been described.^14^ According to the Spanish guidelines for DRESS, TARC/CCL17 has been recommended as a prognostic and diagnostic biomarker since acute DRESS syndrome causes higher serum levels of this protein.^14^ We also evaluated the cases as per the Naranjo scale used to understand the plausible causal relationship between the drug and the ADR, the score of this case was identified as 6 and was categorized as probable ADR, according to the reference Naranjo scale.^15^

**Figure 5.**
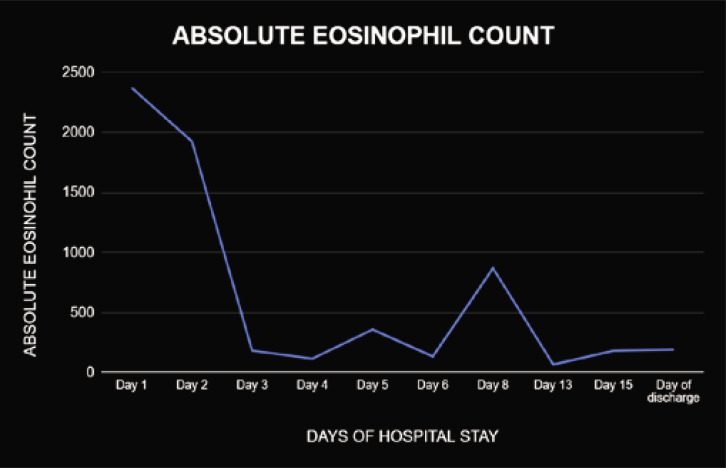
Line chart representing Eosinophilia at different days of hospital stay.

## CONCLUSION

DRESS syndrome must be recognised promptly, and the causative drug must be withdrawn. Indeed, it has been reported that the prognosis is better if drug discontinuation occurs early. Leflunomide, a drug widely used in rheumatology and orthopaedics, must be used with caution since it has the potential to cause DRESS syndrome. Clinical trials must be conducted to identify the most appropriate therapy of DRESS Furthermore, national guidelines have to be developed which will help the clinicians to diagnose DRESS and use the most appropriate therapy.
